# New Therapeutics in Endometriosis: A Review of Hormonal, Non-Hormonal, and Non-Coding RNA Treatments

**DOI:** 10.3390/ijms221910498

**Published:** 2021-09-28

**Authors:** Geraldine Brichant, Ines Laraki, Laurie Henry, Carine Munaut, Michelle Nisolle

**Affiliations:** 1 Obstetrics and Gynecology Department, ULiege, 4000 Liège, Belgium; ines.laraki@student.ulg.ac.be (I.L.); laurie.henry@chuliege.be (L.H.); michelle.nisolle@uliege.be (M.N.); 2 Laboratory of Tumor and Development Biology, Giga-Cancer, ULiege, 4000 Liège, Belgium; c.munaut@uliege.be

**Keywords:** endometriosis, non-coding RNA, cell migration, cell proliferation, apoptosis, fibrosis, angiogenesis, stem cells, inflammation

## Abstract

Endometriosis is defined as endometrial-like tissue outside the uterine cavity. It is a chronic inflammatory estrogen-dependent disease causing pain and infertility in about 10% of women of reproductive age. Treatment nowadays consists of medical and surgical therapies. Medical treatments are based on painkillers and hormonal treatments. To date, none of the medical treatments have been able to cure the disease and symptoms recur as soon as the medication is stopped. The development of new biomedical targets, aiming at the cellular and molecular mechanisms responsible for endometriosis, is needed. This article summarizes the most recent medications under investigation in endometriosis treatment with an emphasis on non-coding RNAs that are emerging as key players in several human diseases, including cancer and endometriosis.

## 1. Introduction

Endometriosis is defined as the presence of endometrial-like tissue outside the uterine cavity. It is a chronic inflammatory estrogen-dependent disease causing pain and infertility in about 10% of women of reproductive age [1,2,3]. Its pathogenesis is complex, and treatment includes medical and surgical aspects.

Medical treatment of endometriosis is based on pain relief but unfortunately none of the drugs can resolve the disease. Hormonal treatment mainly developed by pharmaceutical companies is administered to induce amenorrhea. In a systematic review of the evaluation of medical treatments for endometriosis, Becker et al. estimated that about 10% of patients are not improved by hormonal therapy and require other drugs such as NSAIDs [4]. To date, no optimal medical treatment for endometriosis can be proposed as it frequently fails. The heterogeneity of the distribution of estrogen receptors (ERs) and progesterone receptors (PRs) of the lesions described by Brichant et al. could be the explanation for these results [5]. The development of new biological targets is urgently needed.

Numerous criteria for the ideal medication for endometriosis have been described [6]. Among these criteria, the main interest is to develop curative medications, instead of suppressive medications, that have a simultaneous effect on pain and infertility, a low side effect profile, and safe long-term use and that are non-contraceptive and compatible with pregnancy. In the future, increasing inhibition and avoiding the development of new lesions should be priorities as well as the possibility of treating all phenotypes of endometriosis (superficial peritoneal lesions, ovarian endometriomas, and deep lesions) as well as extrapelvic lesions and adenomyosis, which are frequently associated with endometriosis.

By elucidating the cellular and molecular mechanisms involved in endometriosis, it would be possible to suggest a future candidate pathway for the treatment of endometriosis. The new treatments under investigation that aim at a specific target of the pathogenesis are described in this article. They include new hormonal and non-hormonal treatments such as immunomodulators, antiangiogenic agents, and antifibrotic agents.

## 2. Pathophysiology of Endometriosis

The exact mechanisms responsible for endometriosis are still unclear. The development of endometriosis requires degradation of the extracellular matrix, peritoneal invasion, and the growth of ectopic endometrial stromal and glandular cells [7,8]. The most widely accepted theory is the retrograde menstrual reflux hypothesis [9]. However, while most women have retrograde reflux, only 10–15% of women develop endometriosis. It is assumed that patients with endometriosis have underlying molecular abnormalities that promote the growth of the endometrium outside the uterine cavity. Notably, it seems that ectopic endometrial cells undergo an aberrant adhesion process that could contribute to the development and progression of the disease [10,11]. However, the various mechanisms involved in this aberrant adhesion are still poorly understood. The identification of the different actors involved in the aberrant adhesion of ectopic endometrial cells would be a crucial step in the development of new therapeutic targets.

While the implantation of an ectopic lesion is not hormone-dependent, estrogen plays a key role in cell survival, cell proliferation, and the inflammatory response. As in the eutopic endometrium, ectopic lesions respond to the cyclic secretion of ovarian steroids, mainly estrogen, although they exhibit a significant progesterone resistance [12,13].

Downregulation of ER and restriction of local estrogen production can be achieved through interference of PR signaling with aromatase and 17 beta-hydroxysteroid dehydrogenase type 1 [14]. Progestins negatively influence cell proliferation, inflammation, neovascularization, and neurogenesis in endometriosis. However, PRs are altered in endometriotic lesions with dysregulation of their two isoforms: an increase in PRA (less active); and a decrease in PRB (more active). This progesterone resistance is enhanced by oxidative stress. Compared with other phenotypes, deep endometriosis appears to be more resistant to size regression upon medical treatment.

Ovarian, peritoneal, and deep endometriosis (DIE) are considered to be three different entities [15]. All three can exhibit an alteration in hormonal and immunological function [16]. However, DIE is considered to be more aggressive with higher proliferation activity and less apoptosis. Activins and matrix metalloproteinases are more highly expressed in DIE than in endometriomas and/or peritoneal endometriosis. Fibrosis is another characteristic of DIE.

Targeting specific DIE features, such as fibrosis, is of interest in future treatment research [16,17]. The heterogeneity of the ER-α and PR distribution could explain why endocrine treatments are unable to cure this condition and allow us to only stop progression [5]. Recently, androgen receptors (ARs) were evaluated in stromal and epithelial cells with a higher density in the former than in the latter [18]. A better understanding of the distribution of the steroid receptors, including ERs, PRs, and androgen receptors, in the different types of endometriosis could lead to a better understanding of the pathogenesis of the disease and a targeted treatment.

This review focuses on medical treatments for endometriosis that provide pain relief. Fertility is not discussed.

## 3. Non-Coding RNAs

**Figure 1:**
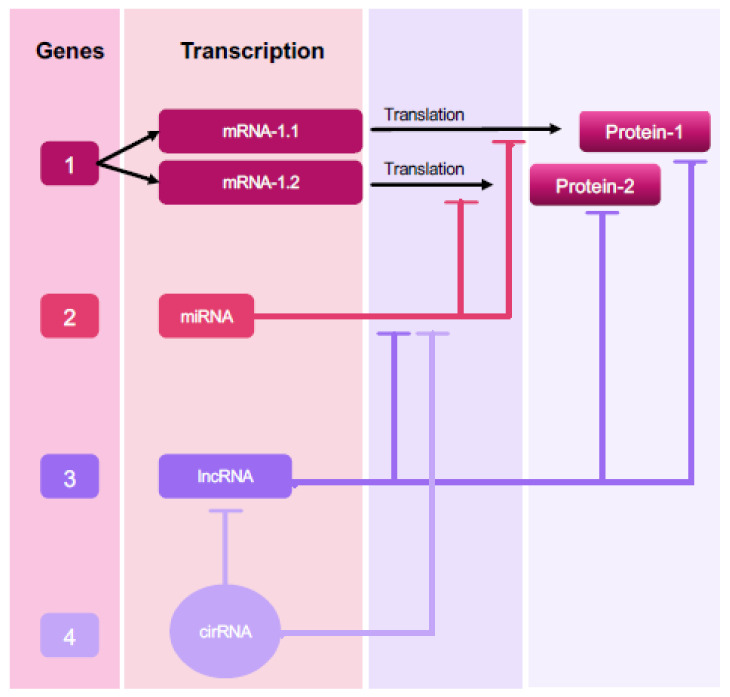
Schematic illustration of the complexity of ncRNA gene networks. In this example, only two splice variants of Gene 1 are translated into proteins. These protein products can be regulated by ncRNAs encoded by Genes 2, 3, and 4, which interact with Gene 1 at the RNA/protein level. Gene 2 encodes miRNAs interacting with mRNAs (mRNA 1.1 and mRNA 1.2). Gene 3 encodes for lncRNAs interacting with the protein products of Gene 1 and acts as a miRNA sponge. Gene 4 encodes for circRNAs serving as a sponge or as a decoy for any RNA-binding event that indirectly regulates Gene 1 products, such as lncRNA or miRNA interactions.

The existence of multiple types of non-coding RNAs (ncRNAs), such as microRNAs (miRNAs), small nucleolar RNAs (snoRNAs), long ncRNAs (lncRNAs), and circular RNAs (circRNAs), has modified our perception of the physiology and development of several diseases, including endometriosis [19,20,21]. They display different regulatory functions that in turn feed into a larger RNA communication network that finally controls the fundamental effector proteins of cellular functions [22,23,24,25]. The important role played by ncRNAs in gene regulation also suggests that they interact with and co-regulate each other [26] (Figure 1).

### 3.1. Insights into ncRNA Classes and Their Association with Endometriosis

Non-coding RNAs have rapidly emerged as key players in several human diseases, including cancer and endometriosis [19,27,28].

**Figure 2:**
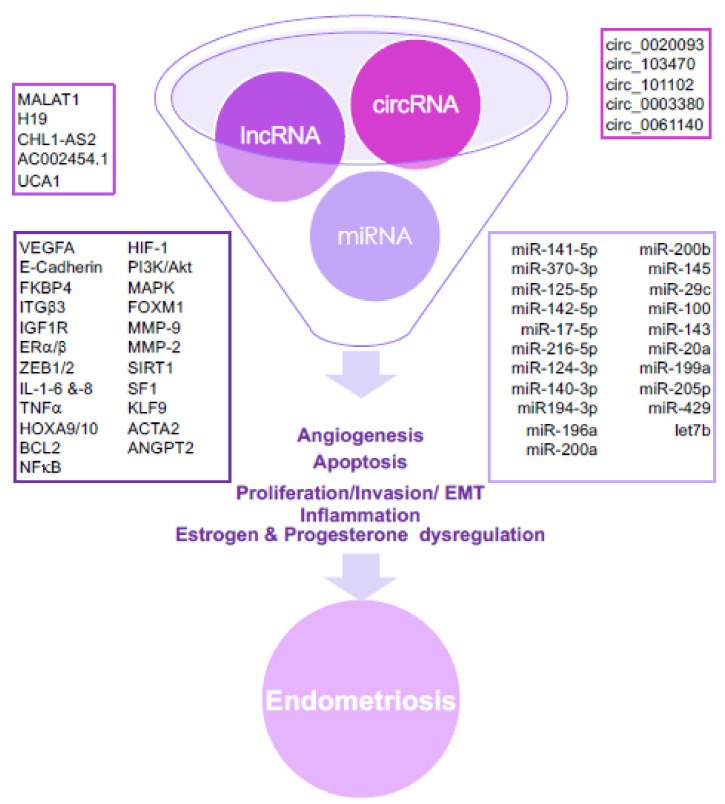
The aberrant expression of several ncRNAs in endometriosis promotes angiogenesis, apoptosis, cell proliferation, cell invasion, cell migration, including the epithelial–mesenchymal transition (EMT), inflammation, and estrogen and progesterone dysregulation. MiRNAs exert their function through modulation of parental gene expression; lncRNAs and circRNAs act as miRNA sponges or RNA decoys, while lncRNAs interact with the protein products of genes and/or act as miRNA sponges.

The use of ncRNAs as a therapeutic tool is still in its infancy even though their ability to be important regulatory molecules makes them attractive targets to silence or overexpress. The fact that the US-FDA has approved three RNAi therapies to date demonstrates the growing success of a technique that has taken 20 years to emerge [29,30,31]. A considerable amount of evidence indicates that ncRNAs potentiate events related to the development of endometriosis, such as angiogenesis, apoptosis, invasion, inflammation, and migration, including the epithelial–mesenchymal transition (EMT), and also estrogen and progesterone dysregulation (Figure 2).

### 3.2. MicroRNAs (miRNAs)

MicroRNAs (miRNAs) are short ncRNAs of about 20 to 24 nucleotides. They regulate gene expression through post-transcriptional repression or degradation of messenger RNA (mRNA) [32,33]. Each miRNA is often able to regulate more than one target and, conversely, mRNAs are frequently targeted by several miRNAs, thus creating a complex network of cooperative regulation [20]. It has been found that miRNAs are altered in eutopic endometrial tissue from women with endometriosis, in eutopic versus ectopic endometrial tissue, and in blood samples from women with or without endometriosis [34,35].

Up- or downregulation of microRNA expression in endometriotic tissue correlates with a dysregulated expression of several target mRNAs relevant to the pathogenesis of endometriosis [36,37].

#### 3.2.1. MicroRNAs and Endometriotic Cell Proliferation

Steroidogenic factor-1 (SF1) regulates the transcription of multiple genes involved in estrogen biosynthesis and is upregulated in endometriosis [38]. Hu et al. showed that miR-370-3p is decreased in the sera and tissue of patients with endometriosis, while SF-1 mRNAs were inversely increased compared with the control groups. Moreover, transfection studies of endometriotic cells by a miR-370-3p mimic or inhibitor altered SF-1 and its downstream target genes (steroidogenic acute regulatory protein (StAR) and CYP19A1). MiR-370-3p is a negative regulator of SF-1 and its upregulation allows for cell proliferation inhibition and apoptosis stimulation in endometriotic lesions. SF-1 downregulation via miR-370-3p could be a new strategy for controlling endometriosis proliferation.

Another possible target could be miR-142-3p/KLF9/Vascular Endothelial Growth Factor-A (VEGF-A) signaling [39]. MiRNA-142-3p targets Krüppel-like factor 9 (KLF9), which mediates autophagy and regulates VEGF-A expression by binding to its gene promoter. MiRNA142-3p is decreased in ectopic endometriotic tissue, while KLF9 and VEGF-A are increased. In vitro studies showed that overexpression of miRNA142-3p or knockdown of KLF9 will decrease cell proliferation and metastasis, increase cell apoptosis, and decrease cell autophagy and vascularization. Finally, intraperitoneal injection of the miRNA-142-3p lentivirus significantly decreased endometriosis lesions in mice.

In 2019, Zhou et al. evaluated miR-205-5p and its target gene angopoietin-2 (ANGPT2) in the ectopic endometrium [40]. Their role in endometriosis progression was confirmed by in vivo and in vitro studies and their expression was correlated to endometriosis stage. ANGPT2 was determined to be a target of miR-205-5p by bioinformatics and luciferase studies. Importantly, the miR-205-5p–ANGPT2 axis was found to activate the ERK/AKT pathway in endometriosis.

MiR-145 inhibits cell proliferation and invasiveness by targeting multiple pluripotency factors, cell adhesion, molecules, and proteolytic factors [41]. Its expression is upregulated in the ectopic compared with eutopic endometrium in patients with ovarian [37] and peritoneal endometriosis [36].

#### 3.2.2. Endometriotic Cell Migration

The miR-200 family, including miR200a, miR200b, miR200c, miR141, and miR429, is one of the most studied groups of miRNAs in endometriosis. These miRNAs could be linked to the pathogenesis of the disease because of their involvement in cell migration and the epithelial–mesenchymal transition (EMT) [42]. Downregulation of several members of the miR-200 family has been reported [36,37,43,44]. They target a complex network of transcription regulators including ZEB1 and ZEB2 (E-box-binding transcription factors 1 and 2, respectively), which are transcriptional repressors for E-cadherin [45].

#### 3.2.3. Progesterone Resistance

Progesterone resistance in the endometrium can decrease endometrial receptivity and increase risks of implantation failure in women of reproductive age with endometriosis [46]. However, the underlying mechanisms responsible for progesterone resistance and impaired implantation have not been thoroughly elucidated [46].

The functional role of miR-29c was examined in vitro by transfection of human endometrial stromal cells with a microRNA mimic and inhibitor for miR-29c [43]. In a baboon model of endometriosis, miR-29c was also found to be upregulated, while the transcript levels of its target, FK506-binding protein 4 (FKBP4), decreased [47]. Combining in vitro and in vivo data, Joshi et al. concluded that compromised FKBP4 expression due to increased miR-29c expression results in impaired progesterone signaling and may contribute to the progesterone resistance observed in women with endometriosis.

The role of miR-196a was also functionally associated with reduced expression of progesterone receptor isoforms in endometriosis [48].

In 2018, Pei et al. evaluated the roles of miR-194-3p in aberrant PR expression and impaired decidualization in endometrial stromal cells (ESCs) from women with mild or minimal endometriosis [46]. They showed through bioinformatics studies that PR expression was directly regulated by miR-194-3p, as its overexpression inhibited ESC decidualization via cellular morphological changes and prolactin levels. MiR-194-3p could therefore play a role in progesterone resistance. Modulating miR-194-3p regulation should be investigated as a future therapeutic strategy for endometriosis.

#### 3.2.4. Endometriosis and Inflammation

Inflammation plays an important role in the development of endometriosis. Sera of patients with endometriosis contain increased levels of miRNA-125b-5p and decreased levels of let-7b-5p [49,50]. Nematian et al. have evaluated the role of let-7b-5p and miRNA-125-5p in the production of proinflammatory cytokines in endometriotic patients [51]. They showed increased levels of TNF-α, IL-1 beta, and IL-6 along with an important upregulation of miRNA-125b and a downregulation of Let-7b in sera of patients with endometriosis compared with controls [51]. Moreover, transfection studies showed a marked upregulation of TNF-α, IL-1 ß, IL-6, and IL-8 in macrophages transfected with miRNA-125b mimics or let-7b inhibitors, suggesting that miRNAs from endometriotic tissue could regulate macrophage-mediated inflammation.

### 3.3. Long ncRNAs (lncRNAs)

LncRNAs represent the largest category of ncRNAs and their relationship to endometriosis has recently been reviewed [52,53]. One outstanding feature of lncRNAs is their ability to regulate gene expression by various mechanisms, one of which is acting as a molecular sponge for miRNAs [54]. One of the first lncRNAs identified, H19, acts as a decoy for several tumor-suppressor miRNAs [55]. In the eutopic endometrium of patients with endometriosis, downregulation of H19 will increase let-7 activity, contributing to a reduction in the proliferation of endometrial stromal cells through Igfr1 expression inhibition [56]. Moreover, downregulation of H19 in ectopic endometrial cells inhibits both cell proliferation and invasiveness and, at the same time, induces an increase in miR-124-3p levels and a decrease in ITGB3 levels [57]. Another study showed that alterations in the lncRNA H19/miR-216a-5p/ACTA2 pathway affect the invasion and migration of eutopic endometrial stromal cells and contribute to fibrosis in women with endometriosis [58]. In a female nude mice model, knockdown of H19 in ectopic endometrial stromal cells (ecESCs) suppressed endometriosis in vivo [59].

The steroid receptor RNA activator (SRA) precursor gene displays multiple splice variants, including the lncRNA SRA and the SRA protein (SRAP) [60]. Low expression levels of the lncRNA SRA and ER-α but relatively high expression levels of SRAP and ER-β were detected in ovarian endometriotic tissues versus normal endometrial tissues. Silencing of the SRA significantly increased ER-α levels but reduced ER-β levels in endometriotic stromal cells (ESCs). The silencing of the SRA also reduced the proliferation and promoted the apoptosis of ESCs [61].

### 3.4. Circular RNAs (circRNAs)

CircRNAs are single-stranded, covalently closed RNA molecules. Unlike traditional linear RNAs, their expression is highly cell-specific, tissue-specific, and developmental-stage-specific. However, similarly to lncRNAs, circRNAs can function as miRNA sponges through the competing endogenous RNAs (ceRNAs). This strongly affects miRNA activity and sequesters miRNAs by binding them to miRNA response elements, thereby increasing or decreasing levels of miRNA target genes [62,63,64]. Currently, little is known about the expression, function, and molecular mechanism of circRNAs in endometriosis.

While functional studies have not yet been conducted to assess their potential therapeutic use, circRNAs through their circular structure have the unique characteristic and advantage of being highly stable compared with the other linear ncRNAS [65].

## 4. Hormonal Treatment

Currently, hormonal treatments remain suppressive and not curative. To date, all hormonal treatments aim at lowering or suppressing local or systemic estrogen levels. The various treatments have similar efficacies, but the tolerance varies between molecules and patients [6]. The first-line medical treatment includes non-steroidal anti-inflammatory drugs and combined oral contraceptives (COCs) or progestins [66]. Second-line treatments include GnRH agonists that reduce estrogen levels to post-menopausal concentrations.

We showed in a retrospective cross-sectional study that women with deep infiltrating endometriosis either undergoing a different hormonal treatment or untreated presented a large intra-lesion, intra-patient, and intra-treatment variance in the expression levels of ERs, which was more evident than any difference between treatments or between treated and non-treated lesions [5]. This heterogeneity in the ER-α and PR distribution could explain why endocrine treatments are unable to cure this condition and allow us to only stop progression.

The most prescribed drugs are COCs and progestins. However, long-term users may complain of irregular bleeding, mastodynia and/or psychological disorders, weight gain, mood swings, or, for some patients, inconvenient androgenic side effects (acne, hair loss, changes in the lipid profile, and/or hirsutism) [67]. Moreover, recently, a higher risk of meningiomas was described, leading to new guidelines in the case of long-term treatment with cyproterone acetate in France [68,69].

Different hormonal therapies are prescribed for patients with endometriosis. One should note that some of the following treatments are off the label for some drug agencies.

### 4.1. Progestins

After binding to the PR, progestins have anti-estrogenic, pro-apoptotic, anti-inflammatory, anti-vasculogenesis, anti-proliferative, and anti-neurogenic effects, all of which allow for pain relief and disease progression inhibition. They reduce or suppress pain in about 90% of patients [70,71]. They stop cellular growth and induce endometriotic decidualization and atrophy [72]. However, one should not forget that endometriosis is well known to be a progesterone-resistant condition [47].

### 4.2. GnRH Agonists

Endometriosis is an estrogen-dependent disease [73]. GnRH agonists (leuprolide acetate, gosereline, etc.), by lowering GnRH pulsatility, repress the gonadotrope axis, preventing estrogen stimulation on ectopic glands. This leads to post-menopausal levels of estrogen, leading to progressive bone loss and/or severe vasomotor symptoms that restrict their use to 6 months without add-back therapy [74]. GnRH agonists also impact endometriosis development through cell migration regulation (see the ‘migration’ section).

### 4.3. GnRH Antagonists

GnRH antagonists (GnRHant) allow for rapid inhibition of gonadotrophin release. A small study evaluating the subcutaneous administration of cetrorelix 3 mg once a week for 8 weeks showed a lower severity of the disease during laparoscopy in 60% of the patients [75]. The main side effects were abnormal uterine bleeding (AUB) and headaches in 20% of the patients. No effect was observed when evaluating mood, hot flushes, libido, or vaginal dryness. Estradiol concentrations remained within the normal range in order to avoid bone loss (50 pg/mL) [76].

Elagolix, an oral GnRHant, allows for gonadotrope inhibition within 24 h after administration [77]. Its efficacy and safety were confirmed in a study of 155 women with endometriosis [78]. Elagolix was evaluated in a double-blind, placebo-controlled study comparing two dosages for 6 months (150 mg and 200 mg die) [79]. Long-term treatment with Elagolix allowed for a significant reduction in dysmenorrhea, non-menstrual pelvic pain, and dyspareunia. The main side effects were hot flushes, and one should note that bone loss and lipids increased after 12 months.

Currently, there is no consensus on the length of a GnRHant treatment.

### 4.4. Dopamine Agonists

Most treatments aim to produce a hypoestrogenic environment. However, when patients wish to conceive, maintaining normal ovarian function is mandatory. Vascularization is one of the main targets in the development of endometriosis. Aiming at angiogenesis is a promising treatment. Besides VEGF inhibitors (e.g., bevacizumab), endogenous angiogenesis inhibitors, statins, COX-2 inhibitors, PPAR-gamma, and dopamine agonists (e.g., bromocriptine, cabergoline, and quinagolide) are promising molecules [6,80,81]. In an experimental model, dopamine agonists (DAs) blocked cellular endothelial and endometrial proliferation by modulating pro- and anti-angiogenic pathways, reducing the endometriotic lesion size [82]. This seems to depend on impaired VEGF secretion and inactivation of its type 2 receptor. DAs also reduce nerve fibers’ density. In human studies, quinagolide reduced pain and endometrioma size.

Plasminogen inhibitor-1 (PAI-1) is a gene encoded by serpin-1. DAs decrease serpin-1, reducing PAI-1. Pentoxifylline, a PAI-1 inhibitor, could increase pregnancy rates in women with endometriosis, although the evidence is of low quality [83]. Combining DAs that reduce the endometriosis lesion size while allowing for ovulation and pentoxifylline could be a novel approach to the medical treatment of endometriosis [81].

The main advantages of DAs are their safety and the fact that they do not interfere with ovulation. However, as long-term treatment with cabergoline and pergolide is associated with cardiac insufficiency, non-ergot-derived dopamine agonists should be preferred in endometriosis treatment.

### 4.5. Selective Estrogen Receptor Modulators

In a mouse model of endometriosis, the efficiency of the selective estrogen receptor modulator (SERM) bazedoxifene (BZA) combined with a conjugated estrogen in a tissue-selective estrogen complex (TESC) was evaluated. The effect on the regression of lesions can be explained by the negative effect of BZA on the endometrium [84,85]. BZA exerts a powerful antagonistic effect on estrogen receptors in the endometrium. In addition, Wang et al. demonstrated that bone-marrow-derived stem cells (BMDSCs) migrate to the endometrium via the CXCL12–CXCR4 signaling pathway [86]. Endometrial stromal cells produce the chemokine CXCL12, while BMDSCs express the CXCR4 receptor. Estradiol, which is increased locally in endometriosis, increases the production of both CXCL12 and CXCR4. Therefore, the inhibition of estradiol receptors by the TESC blocks the migration of BMDSCs to the endometrium. This can also be achieved by using CXCR4 antagonists [87].

Raloxifene is another SERM that was evaluated in the treatment of endometriosis [88]. Unlike BZA, raloxifene did not induce a reduction in the lesions and, moreover, in a placebo-controlled study evaluating pain in patients receiving or not receiving raloxifene after surgical excision of endometriotic tissue, treated patients experienced pain sooner than the placebo arm [89]. The reason evoked for this discrepancy was that raloxifene decreases epithelial cell proliferation but has no effect on the stromal cells that are the major component of endometriotic lesions [90]. Lastly, estradiol agonism and antagonism also influence nociceptors and the perception of pain.

### 4.6. Selective Progesterone Receptor Modulators

Selective progesterone receptor modulators (SPRMs) are synthetic steroids that have both antagonist and agonist properties when binding to progesterone receptors [91]. They induce amenorrhea with no hypoestrogenic side effects and allow for a reduction in uterine bleeding [92,93]. Ulipristal acetate (UPA), asoprisnil, and mifepristone act primarily as progesterone antagonists. UPA reduced the size of lesions, decreased cell proliferation, and induced apoptosis in a mouse model of endometriosis [94,95]. Mifepristone reduced the size of lesions in both human and mouse models [96,97]. Despite promising results, the long-term effects and side effects of SPRMs remain to be evaluated.

## 5. Non-Hormonal Treatment

### 5.1. Angiogenesis

Angiogenesis is defined by the formation of new blood vessels that sprout from the primary vascular network and is a prerequisite for the maintenance and growth of endometriosis [98]. Angiogenesis is essential for normal reproduction and endometrial growth and remodeling [99]. Treatments targeting angiogenesis, besides the previously described DAs, could therefore be of interest in endometriosis.

The angiogenic inhibitor caplostatin (a non-toxic TNP-470 inhibitor) and endostatin-delayed luciferase signaling produced a reduction in angiogenesis in a mouse model of endometriosis [100].

Tissue factor is not normally expressed in normal endothelial cells but plays a role in aberrant embryonic and oncological angiogenesis [101]. The immunoconjugate molecule Icon binds with high affinity and specificity to aberrant endothelial tissue factor, inducing a cytolytic immune response that allows for devascularization and downsizing of pre-established lesions [101]. Animal studies in athymic mice have confirmed the regression of pre-established lesions. In a baboon model, Hufnagel et al. showed a reduction in red, hypervascularized lesions but no effects on blue or white lesions or on adhesions [102].

VEGF-A is the most important angiogenic cytokine expressed by the proliferative endometrium [98]. VEGF is strongly expressed in endometriotic lesions, particularly red lesions, but is also expressed in peritoneal macrophages. It has been demonstrated in in vitro and in vivo studies that the VEGF soluble receptor prevents the formation of new lesions by binding to VEGF receptor 1 [103]. The VEGF soluble receptor could be used to delay recurrence. Hull et al. showed that soluble fms-like tyrosine kinase 1 (sFlt-1) and an antibody targeting VEGF (two VEGF-A antagonists) were able to significantly inhibit lesion growth [104] in an experimental sponge implantation and mice model.

The monoclonal antibody bevacizumab was studied in a rat model and found to reduce microvessels’ density; however, its adverse effects (dry mouth, cough, loss of appetite, diarrhea, vomiting, headache, cold symptoms, hair loss, changes in the sense of taste, loose teeth, hypertension) make it an unacceptable treatment for a benign disease [105].

Galectins (Gals) are glycan-binding proteins that bind specifically to β-galactosides [106]. Gal-3 is involved in angiogenesis, embryogenesis, cell adhesion, differentiation, and proliferation, apoptosis, mRNA splicing, and regulation of the immune system response and is more strongly expressed in tumoral endothelial cells than in cells of normal endothelial tissue [107]. Gal-3 increases GAL-3, VEGF, and VEGFR-2 expression and induces a shift towards type 2 macrophages (M2), which are responsible for the release of pro-angiogenic factors. Gal-3 is overexpressed in peritoneal and deep infiltrating endometriosis and in the eutopic endometrium of women with endometriosis compared with the eutopic endometrium of women without endometriosis [108]. Gal3C (a carbohydrate recognition domain of recombinant galectin-3) inhibits Gal-3 and leads to a regression in lesion size but also a decrease in angiogenic markers, the vascular density, and the macrophage population [107]. In addition, Gal3 treatment also reduces the expression of TGFß1, which promotes a decrease in VEGF levels and the polarization of macrophages towards the M1 phenotype. Administration of monoclonal antibodies targeting galectin-1 also significantly reduces the size of endometriosis lesions and the development of new blood vessels [106].

In conclusion, numerous anti-angiogenic molecules have been evaluated in the treatment of endometriosis and have shown promising results in terms of the regression of hypervascularized lesions. However, they have shown few to no effects on fibrotic lesions and adhesions. Although numerous anti-angiogenic drugs have been studied in vitro, in vivo clinical trials are needed in order to evaluate their safety and efficiency. More studies are needed before these drugs can be routinely applied to treat endometriosis in humans.

### 5.2. Fibrosis

An inflammatory reaction associated with endometriosis is responsible for the secretion of cytokines by leucocytes, which stimulate the proliferation and activation of myofibroblasts. Activated myofibroblasts secrete pro-inflammatory cytokines, recruit inflammatory cytokines, and amplify the fibrotic reaction. Many studies have demonstrated the important role that the immune system plays in the progression of endometriosis. Macrophages play a central role in the orchestration of inflammation and fibrosis in endometriosis. A possible role of B lymphocytes in fibrosis development has been suggested in a study comparing severe combined immunodeficiency (SCID) mice and NUDE mice (which do not have their B cells deleted) [109]. Limited fibrosis was observed in SCID mice compared with NUDE mice, in which fibrosis surrounding the endometriotic implant was noted.

Very recently, ibrutinib, a selective covalent and irreversible inhibitor of Btk, a non-receptor kinase essential for B cell development and the function of mature B cells, was found to be associated with a reduction in the size and activity of endometriotic lesions in a mouse model [80]. The expression of inflammatory and fibrotic markers was also decreased. Btk inhibitors have shown anti-tumor activity in animal models and in the clinic [110]. In in vitro studies, the signaling and metabolism of the bioactive sphingolipid sphingosine1 phosphate (S1P), which is involved in inflammation and the immune response, were altered in endometriosis and SIPR expression highlighted ovarian and deep endometriotic lesions [17]. S1P mediates TGFß1 fibrosis induction and modulation of S1P signaling could represent an innovative pharmacologic target for endometriosis.

Transcription factor 21 (TCF 21) and periostin are involved in the regulation of fibrosis in endometriosis [111]. Under normal conditions, TCF 21 is activated only after tissue injury. It induces periostin production, which contributes to fibrosis and the activation of immune cells to produce proinflammatory cytokines. This results in aberrant expression of TCF 21. TCF 21 could be a key regulator for switching off periostin production and the progression of the disease.

Heparin was used as an agent for the treatment of endometriosis-associated fibrosis as in vitro it was found to decrease the expression of myofibroblasts in a collagen gel structure system [112]. To date, this has not been confirmed in a clinical trial.

In conclusion, the cellular and molecular actors in fibrosis have been identified; however, to date, no therapeutic agent has been evaluated in clinical studies.

### 5.3. Inflammatory Cytokines and Immune Cells

Inflammatory cytokines are involved in the pathogenesis of endometriosis. The role of interleukin (IL)-1/IL-33 signaling in the development of endometriosis and the effect of the Il-1R-associated kinase (IRAK4, a downstream signal of MyD88) inhibitor have been studied in a mouse model [113]. IL-1 is involved in the development of endometriosis and MyD88 signaling is essential to the growth of endometriotic lesions. Oral administration of an IRAK4 inhibitor reduced the proliferation of epithelial cells and the growth of lesions.

Anti-TNF-α drugs can inhibit the inflammation process and are used to treat other inflammatory conditions. Lu et al. demonstrated that there is insufficient evidence to support the use of anti-TNF-α drugs in endometriotic women suffering from pelvic pain despite preliminary studies [114]. In a Cochrane review evaluating 334 infertile endometriosis patients, Liu et al. also concluded that there was insufficient evidence to recommend pentoxifylline, a competitive phosphodiesterase inhibitor with immunomodulatory properties [115].

### 5.4. Stem Cells

Stem cells are undifferentiated cells that can differentiate into many cell types and have a significant regenerative capacity. The role of BMDSCs in the regeneration of the endometrium was first demonstrated in 2004 [116]. The authors showed that donor-derived (HLA-discordant) cells were detected in an endometrial biopsy of women that underwent a HLA-mismatched bone marrow transplant, allowing for determination of the origin of the cells. This was first confirmed in a mouse model of endometriosis in which female mice underwent a male bone marrow transplant. After the bone marrow transplant, male BMDSCs were found in the endometrium [117]. Thus, BMDSCs could play a role in the development of distant endometriosis implants. In a second model, mice underwent an endometrial implantation in their peritoneal cavity as well as a bone marrow transplant from LacZ transgenic mice. Finally, cells expressing LacZ were found in the ectopic endometrium of transplanted mice and hysterectomies [117]. All these data confirm the involvement of BMDSCs in the development of endometriosis. However, the mechanisms by which these BMDSCs differentiate into endometrial cells have not yet been fully elucidated. It seems that the peritoneal microenvironment plays a role in facilitating their development via immunological, genetic, and environmental factors but also paracrine effects.

Endometrial stem cells (EnSCs) have been identified in the eutopic and ectopic endometrium but also in the menstrual blood of patients with endometriosis [118]. EnSCs play a role in angiogenesis and immunomodulation, suggesting that they may be responsible for the impaired angiogenesis and immunomodulation found in endometriosis patients. Furthermore, the EnSC populations of endometriosis patients differ significantly from those of healthy patient populations. Indeed, ectopic EnSCs have a greater capacity for invasion and angiogenesis than their eutopic analogues; they also present a different immune phenotype with upregulation of toll-like receptors, collectin, pro-inflammatory factors, migration factors, and angiogenic factors, thus favoring the migration, proliferation, and invasion of endometriosis lesions and enhancing the degree of fibrosis in these same ectopic lesions.

Eight signaling pathways implicated in the differences between eutopic and ectopic populations of EnSCs were identified [119].They include microRNAs of signaling pathways in cancer, complement and coagulation cascades, cytokine–cytokine receptor interactions, ECM–receptor interactions, focal adhesion, and ovarian steroidogenesis. They are probably mediated by HIF-1, PI3K-Akt, and TGF-ß [119]. Determining these different factors may help to develop new therapeutic targets for the treatment of endometriosis.

Chen et al. suggest that endometriosis promotes the differentiation of BMDSCs into stromal, epithelial, or leukocyte cells through paracrine signalization but also increases the expression of programmed cell death 1 (PD-1) [120]. Increased expression of PD-1 in immune cells and the PDL1 ligand probably promotes the growth of ectopic endometrial cells by inhibiting T cells and leading to local immune tolerance. Therefore, therapies that antagonize PD-1 by lifting the immunosuppression induced by PD-1 could be interesting strategies. In addition, CXCL12 inhibits the expression of PD-1 and plays a role in the modulation of the immune system via the migration, development, and survival of lymphocytes [120]. A study on a mouse model of endometriosis by Ersoy et al. compared the effects of MPA (a synthetic progestin), letrozole (an aromatase inhibitor), and leuprolide acetate (a GnRH agonist) on the reduction in lesion size and recruitment of BMDSCs [121]. All three treatments resulted in a reduction in the lesion size. However, treatment with letrozole and leuprolide acetate led to a greater reduction in the size of the lesions and to a reduction in the recruitment of BMDSCs, giving these two molecules a longer lasting effect than synthetic progestins. Again, this can be explained by the increase in production of CXCL12 and CXCR4 due to the local increase in E2 levels [121]. Both letrozole and leuprolide acetate are effective at reducing E2 levels, while progestins will have no effect on the production of CXCL12 and CXCR4 and, therefore, on the recruitment of BMDSCs. Thus, therapies aimed at reducing the local production of E2 can reduce the recruitment of these BMDSCs and so reduce the growth and development of endometriosis lesions.

In conclusion, these data lead us to suppose that stem cells are involved in the pathogenesis of endometriosis, particularly for lesions at some distance from the pelvic cavity. They therefore represent an interesting therapeutic target in order to limit the growth and development of lesions. However, further studies on the signaling pathways to specifically target are necessary.

### 5.5. Migration

Tensin 1 (TNS1) is a protein found in deep adhesions and could play a role in the migration of endometrial cells [10]. TNS1 is involved in cell survival, apoptosis, angiogenesis, inflammation, and hypoxia. Previous studies have shown an overexpression of TNS1 in the ectopic endometrium of patients with endometriosis. Rahmawati et al. evaluated the effect of GnRH agonists on the expression of this protein in the serum and endometrium of patients with endometriosis [122]. They showed that the expression of the TNS1 protein and mRNA was decreased in patients treated with GnRH agonists, and they also observed a decrease in TNS1 in the serum of the patients. Thus, the hypoestrogenic environment induced by GnRH agonists could explain the effects on TNS1 expression, both in endometriosis lesions and in the serum of patients, and this would therefore make it possible to block the migration and adhesion of these ectopic cells and in fine the progression of the disease. Finally, TNS1 could be of medical interest as a potential therapeutic target in endometriosis but also as a biomarker to assess the efficacy of GnRHa treatments. However, increased expression of TNS1 has been identified in ovarian endometrioma but not in other endometriosis localizations. Thus, it is possible that the aberrant expression of the adhesion molecules varies according to the phenotype of the pathology [10]. Further studies on larger cohorts are therefore necessary in order to determine the exact role of TNS1 in the progression of endometriosis and the mechanisms by which GnRHa induces a decrease in the expression of TNS1.

### 5.6. Apoptosis

Retinoic acid is a powerful modulator of transcription genes involved in cell growth, differentiation, and apoptosis [123]. The absorption, metabolism, and activity of retinoic acids are reduced in endometriosis implants, weakening estrogen metabolism and increasing resistance to apoptosis. The retinoic acid signaling pathway is a promising new target for the treatment of endometriosis. Fenretinide is a synthetic retinoid analogue that promotes apoptosis and is less toxic than other retinoids. Pavone et al. evaluated the effect of fenretinide treatment on endometrial cells in vivo and demonstrated that it significantly reduced the total number of endometrial cells and the expression of nuclear antigens. Fenretinide also reduces aromatase activity in vitro. However, retinoids are teratogenic molecules, so their use in patients of childbearing age requires a combination with an effective contraceptive [123].

Endometriosis is associated with increased oxidative stress, inflammation, and deregulation of autophagy. Among the antioxidant defenses, nuclear erythroid factor 2 (Nrf2) is a transcription factor involved in the upregulation of various antioxidant enzymes, such as quinone oxidoreductase (NQO1), glutamate cysteine ligase (GCL), and heme oxygenase-1 (HO-1) [2]. The expression of the Nrf2 and GCL genes is reduced in the ectopic endometrium of patients with endometriosis. The activity of the mammalian target of rapamycin (mTOR), a potent inhibitor of autophagy, is significantly increased in the ectopic endometrium of patients with endometriosis. Quercetin is a natural flavonoid contained in various fruits and vegetables. Quercetin has anti-inflammatory, antioxidant, anti-tumor, anti-diabetic, cardioprotective, and hepatoprotective activities. Quercetin can increase the autophagy and apoptosis of cancer cells by inhibiting the Akt, mTOR, and 1α signaling pathways induced by hypoxia. Metformin is a widely used anti-diabetic drug that has been shown to be effective in various diseases, such as polycystic ovary syndrome, cancer, and cardiovascular disease. The numerous effects of metformin appear to be mainly related to the activation of the AMP-activated protein kinase (AMPK), a regulator of cellular energy homeostasis and an mTOR inhibitor. The size and architecture of endometriotic lesions in a rat model were evaluated after treatment with quercetin and/or metformine [2] as well as adhesions and serum levels of estradiol, progesterone, and TNF-α, markers of oxidative stress and autophagy [123]. Serum levels of TNF-α and 17ß-estradiol were significantly increased in rats with endometriosis; however, the enzymatic activity of quinone oxidoreductase (NQO1) and the levels of expression of nuclear erythroid factor 2 (Nrf2) and autophagy markers were decreased. In addition, the gene expression of the mammalian target of rapamycin (mTOR) was increased in ectopic endometrial tissues compared with their eutopic homologues [2]. Treatment with quercetin and metformin significantly reversed these alterations but also decreased the size of endometrial implants and the expression levels of mTOR and autophagic markers in the ectopic endometrium. Treatment with quercetin and metformin, alone or in combination, can reduce serum E2 levels. The effect of metformin on the decrease in E2 levels is probably related to the suppression of aromatase activity in the stromal cells of the ectopic endometrium. In addition, unlike other conventional treatments, quercetin and metformin have no effect on the physiological production of estrogen and progesterone.

All these elements, therefore, make quercetin and metformin potential treatments for endometriosis. To date, no study has evaluated their effects on endometriosis in mammals or humans.

Resveratrol is a natural polyphenol with anti-proliferative and anti-inflammatory properties. Increasing evidence suggests that it has antineoplastic, anti-inflammatory, and antioxidant properties as well as cell growth inhibition and apoptosis induction activities [124]. Resveratrol is currently widely used as a dietary supplement but has not yet been approved by the FDA for a specific clinical application and its long-term safety remains to be determined. Treatment with resveratrol resulted in a reduction in both the number and size of endometriosis implants and a reduction in the invasion of human endometrial stromal cells in a mouse model. In addition, endometriosis lesions exposed to resveratrol showed an increase in apoptotic activity and a decrease in the invasion capacity of human endometrial cells. However, the precise mechanisms by which resveratrol interacts with endometrial cells are still unknown. Further studies are necessary to evaluate the effects of resveratrol on the endometrial tissues of patients with endometriosis.

## 6. Conclusions

Endometriosis is a chronic benign disease that impacts the lives of about 10% of women of reproductive age. To date, treatments have been palliative in nature and the disease often recurs after medication is stopped. Numerous molecules targeting the angiogenesis, fibrosis, inflammation, migration, apoptosis, and signaling processes involved in endometriosis are currently under investigation. While most of them have yielded good results in vitro, extended in vivo studies are still needed. Non-coding RNAs are emerging as key factors in the development of human diseases such as cancer and play a role in the pathogenesis of endometriosis. Several miRNAs are involved in endometriotic cell migration, proliferation, and proliferation resistance. Specifically aiming at the latter could be of utmost interest in order to ease patients’ symptoms without major secondary effects.
